# High glucose and TGF-β1 reduce expression of endoplasmic reticulum-resident selenoprotein S and selenoprotein N in human mesangial cells

**DOI:** 10.1080/0886022X.2019.1641413

**Published:** 2019-09-05

**Authors:** Fumeng Huang, Yuanxu Guo, Li Wang, Lanmei Jing, Zhao Chen, Shemin Lu, Rongguo Fu, Lifang Tian

**Affiliations:** a Department of Nephrology, The Second Affiliated Hospital of Xi’an Jiaotong University, Xi’an, China;; b Department of Genetics and Molecular Biology, Xi’an Jiaotong University College of Medicine, Xi’an, China

**Keywords:** Endoplasmic reticulum, selenoprotein S, selenoprotein N, HMCs, glucose, TGF-β1

## Abstract

There are seven endoplasmic reticulum (ER)-resident selenoproteins in human body and they can regulate the inflammation, oxidative stress, and ER stress. We established transforming growth factor-β1 (TGF-β1) or high glucose (HG) induced human mesangial cells (HMCs) fibronectin expression model *in vitro*. Next, the expression changes of seven ER-resident selenoproteins were detected under HG conditions and we found selenoprotein S (SELENOS), selenoprotein N (SELENON) were significantly down-regulated but selenoprotein M was significantly up-regulated in transcription level. Furthermore, we found that TGF-β1 and HG down-regulated the expression of SELENOS and SELENON in a time- and dose-dependent manner, respectively. Finally, SELENOS was knocked down by siRNA and we found that knocking down SELENOS decreased TGF-β1 induced fibronectin expression. Our research indicates the potential value of ER-resident selenoproteins on renal fibrosis.

## Introduction

Renal fibrosis is the common pathway and final outcome of various progressive chronic kidney disease including diabetic nephropathy. The key pathological change of diabetic nephropathy is proliferation and hypertrophy of mesangial cells, excessive accumulation of extracellular matrix (ECM) proteins which eventually leads to nodular glomerulosclerosis [1,2]. Mesangial cells are the intrinsic cells of the glomerulus. They can produce ECM, secrete fibronectin, phagocytose, remove foreign bodies and regulate capillary blood flow in the glomerulus [3]. In pathological conditions, high expression of fibronectin leads to renal fibrosis. Oxidative stress and endoplasmic reticulum (ER) stress are important factors to the ECM glomerular pathology in diabetic nephropathy [4,5] and in unilateral ureteral renal fibrosis mouse model or kidney cells stimulated by transforming growth factor-β1 (TGF-β1) [5,6]. Hyperglycemia in diabetic nephropathy generates elevated levels of reactive oxygen species (ROS) which induce kidney damage. Both high glucose (HG) and TGF-β1 could induce the production of ROS in mesangial cells [6,7].

Selenoproteins refer to a class of proteins with selenocysteine in their peptide chains. There are 25 selenoproteins in humans [8], which play important roles in a variety of physiological and pathological processes, such as regulation of redox homeostasis [9], immune function [10], and thyroxine synthesis [11]. Among them, there are seven ER-resident selenoproteins, including selenoprotein 15 (SELENOF), deiodinase 2 (DIO2), selenoprotein S (SELENOS), selenoprotein N (SELENON), selenoprotein K (SELENOK), selenoprotein M (SELENOM), and selenoprotein T (SELENOT) [12]. ER-resident selenoproteins are considered to be involved in the regulation of inflammation, oxidative stress, and ER stress [13].

To date, only two of them have been involved in human disease research, namely SELENOS and SELENON. However, there are few reports on the role and mechanism of ER-resident selenoproteins in renal diseases. Whether those ER-resident selenoproteins influence the progression of glomerular sclerosis and tubulointerstitial fibrosis is unclear. Here, we hypothesized that ER-resident selenoproteins play a role in the HG or TGF-β1 induced pathologic change of mesangial cells. Therefore, we established the cell model of HG or TGF-β1 induced the expression of fibronectin which is a marker for renal fibrosis. Furthermore, the expression change of seven ER selenoproteins was observed. Finally, we set out to investigate the effect of SELENOS on TGF-β1 induced fibronectin expression.

## Materials and methods

### Reagents

The RPMI-1640 medium was purchased from Hyclone (Logan, UT, USA) and fetal bovine serum from Gibco (Carlsbad, CA, USA). Recombinant human TGF-β1 protein was purchased from the Sino Biological Company (Wayne, PA, USA) and the glucose from Sigma (St. Louis, MO, USA). The Trizol reagent, reverse transcription kit and SYBR Green System were purchased from Roche Company (Basel, Switzerland). The primary antibody against β-actin (CST, 4970) was purchased from Cell Signaling Technology (Danvers, MA, USA), primary antibody against SELENOS (Abcam, Cambridge, UK, 223721) or fibronectin (Abcam, Cambridge, UK, 32419) from Abcam (Cambridge, UK), primary antibody against SELENON (Novus, Saint Charles, MO, USA, 34098) from Novus (Saint Charles, MO, USA). The secondary horseradish peroxidase-coupled anti-rabbit antibodies (CST, 5571) were purchased from Cell Signaling Technology (Danvers, MA, USA).

### Cell line and cell culture

Human mesangial cells (HMCs) were purchased from the Type Culture Collection of the Chinese Academy of Sciences (Shanghai, China). Cells were maintained in growth media (1640 medium with 10% FBS, 2% penicillin/streptomycin) without any supplemental selenium at 37 °C in a 100% humidified atmosphere of 5% CO_2_ and 95% air (standard culture condition). The medium was refreshed every two days. Once confluent to 80–90%, cells were seeded in a 12-well plate at a concentration of 2 × 10^5^ per well for further experiments.

### HMCs treatment with HG or TGF-β1

HMCs were seeded in a 12-well plate at a concentration of 2 × 10^5^ per well for 24 h. Next, after 12 h serum-free treatment, TGF-β1 (0–10 ng/mL) or different dose (11.1–30 mM) of glucose was used to stimulate the cells for appropriate time.

### Transient transfection of negative control sequence or siRNA sequence against SELENOS

When cells were confluent to 50–70% in the 12-well plate, they were refreshed with 900 μL serum-free medium. Then, 50 nM negative control (NC) sequence or 50 nM siRNA mixture sequence against SELENOS was transfected into the cells by lipofectamine 2000. The siRNA sequences against SELENOS are listed in Table 1.

**Table 1.** Small interfering RNA sequences.

Gene	Sense	Antisense
Negative control	5′-UUCUCCGAACGUGUCACG-3′	5′-ACGUGACACGUUCGGAGAATT-3′
SELENOS-582	5′-GGAGGUUAUAACCCGUUGUTT-3′	5′-ACAACGGGUUAUAACCUCCTT-3′
SELENOS-195	5′-CCACCUAUGGCUGGUACAUTT-3′	5′-AUGUACCAGCCAUAGGUGGTT-3′
SELENOS-308	5′-GCUGUGGAACCUGAUGUUGTT-3′	5′-CAACAUCAGGUUCCACAGCTT-3′

### RNA extraction and real-time PCR

Total RNA was extracted using a Trizol Kit. RNA purity and content were determined with a UV/visible spectrophotometer. Synthesis of cDNA, using 2 μg of total RNA, was conducted with a Roche reverse transcription kit according to the manufacturer’s instructions. Quantitative gene expression analysis was performed with real-time PCR on an iQ5 Real-Time PCR System (BIO-RAD, Hercules, CA). A total of 0.4 μL of cDNA was used in 20 μL of the reaction mixture, and the reaction program was as follows: 95 °C for 30 s, followed by 40 cycles at 95 °C for 5 s, 60 °C for 15 s, 72 °C for 30 s, and finally, one cycle at 72 °C for 30 s. Data were normalized by β-actin RNA expression. The mRNA copy numbers were calculated with the instrument software using the Ct value. The forward and reverse primers sequences are listed in Table 2.

**Table 2.** Real-time PCR primer sequences.

Gene	Forward primer	Reverse primer
β-Actin	5′-AGTTGCGTTACACCCTTTCTTG-3′	5′-TCACCTTCACCGTTCCAGTTT-3′
Sel 15	5′-CCTGATTGCAGAGGATGCTG-3′	5′-AGGGTCTGAACCACGGACAT-3′
DIO2	5′-GGAAGATCGATGTGCAGCAG-3′	5′-GCCGGACTTCTTGAAGGTTG-3′
SELENOT	5′-CGGTGGAGTTGCCACTGTAG-3′	5′-TAGGCCAGCTCCACCTCTTC-3′
SELENOK	5′-AAGTGTTGGACAGCCGGAGT-3′	5′-CTAGGGCCACGCAGATGATT-3′
SELENOM	5′-TGACAGCTGAACCGCCTAAA-3′	5′-TCCTGCACTAGCGCATTGAT-3′
SELENON	5′-AGCCAAGGCTGAGAACAAGC-3′	5′-GGGACGAGTTCTCCTGGTTG-3′
SELENOS	5′-AAGAAGCCCCAGGAGGAAGA-3′	5′-CCCTTGGTCAAGAAGCAACC-3′

### Western blotting

Total protein samples were isolated in lysis buffer, and the protein contents were determined by BCA assay. Proteins were separated on 8–12% SDS-PAGE gels according to the molecular weight and then transferred onto a nitrocellulose membrane. After 2 h blocking, the membranes were incubated with primary antibodies followed by secondary antibodies. Protein bands were visualized on X-ray film (Kodak, Rochester, NY) using an ECL reagent.

### Statistical analyses

The results were expressed as the *n*-fold increase with respect to the controls as the mean ± SEM. Statistical analysis was performed with the Mann–Whitney *U* test or ANOVA followed by the Kruskal–Wallis test for multiple comparisons. Statistical significance was concluded for values of *p*<.05. These analyses were performed with SPSS 15.0 statistical software (SPSS Inc., Chicago, IL).

## Results

### High glucose and TGF-β1 promote the expression of fibronectin in HMCs

**Figure 1.**
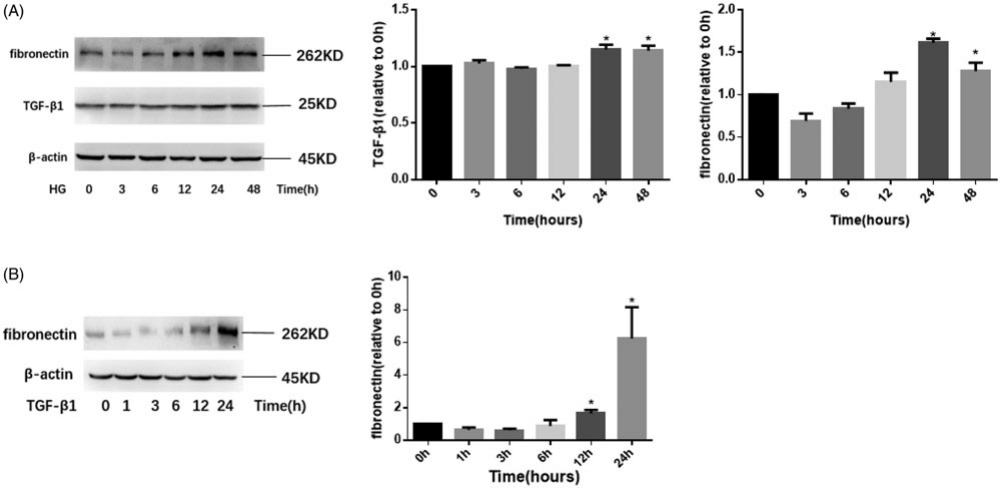
HG and TGF-β1 promote the expression of fibronectin in HMCs. HMCs were stimulated with HG (30 mM glucose) or TGF-β1 (10 ng/mL) for different time points. Then, cell total protein was extracted and the expression of TGF-β1 or fibronectin was performed by western-blotting. (A) The expression of TGF-β1 and fibronectin under the stimulation of HG at indicated time points (0, 3, 6, 12, 24, and 48 h). (B) The expression of fibronectin under the stimulation of TGF-β1 at indicated time points (0, 1, 3, 6, 12, and 24 h). HG: high glucose. *n* = 3, *p<.05 versus 0 h group.

To determine the time points at which TGF-β1 or HG stimulates the increase of ECM in HMCs, cells were stimulated with TGF-β1 (10 ng/mL) or HG (30 mM) for different times after 12 h synchronization. Then, total cell protein was extracted for western-blotting test. As shown in Figure 1(A), after stimulation with 30 mM glucose for 24 h, the expression of TGF-β1 and fibronectin began to be up-regulated and another 24 h stimulation could not further increase the expression level. Similarly, as shown in Figure 1(B), TGF-β1 alone induce the expression of fibronectin in a time-dependent manner, and the expression level peaked at 24 h. The above results show that TGF-β1 and HG could induce the expression of fibronectin independently.

### The expression of HMCs ER-resident selenoproteins under HG stimulation

**Figure 2.**
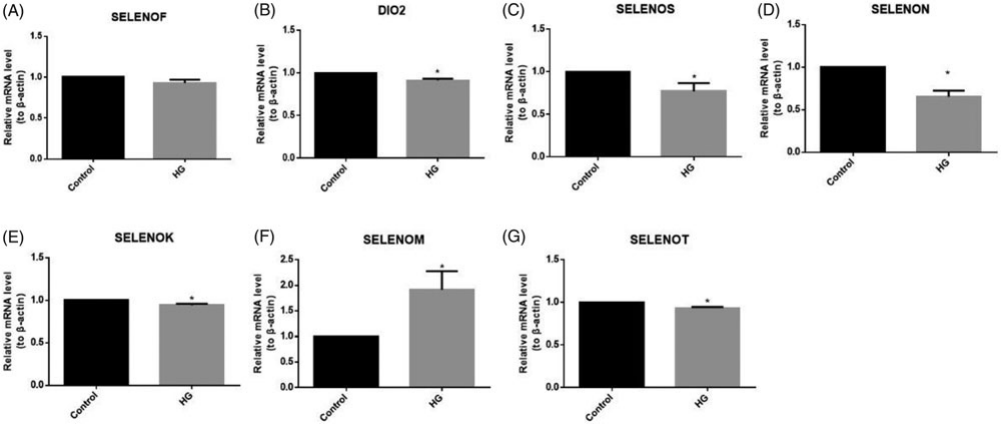
The expression of HMCs ER-resident selenoproteins under HG stimulation. HMCs were stimulated with HG (30 mM glucose) for 24 h. Total RNA was extracted and the relative expression level of ER-resident selenoproteins was performed by real-time PCR. The transcriptional expression level change of (A) Sel15, (B) DIO2, (C) SELENOS, (D) SELENON, (E) SELENOK, (F) SELENOM, and (G) SELENOT after the stimulation of HG. HG: high glucose. *n* = 3, *p<.05 versus control group.

In order to investigate whether ER-resident selenoproteins play a role in the HG or TGF-β1 induced HMCs pathological change, we set out to test the expression level of ER-resident selenoproteins under the condition of HG stimulation. After 12 h synchronization, cells were stimulated with glucose (30 mM) for 24 h hours and then total RNA was extracted for real-time PCR test. As shown in Figure 2, the expression of SELENOF (Figure 2(A)) has no significant change under the HG condition. SELENOM (Figure 2(F)) expression is significantly up-regulated by glucose. The expression level of DIO2 (Figure 2(B)), SELENOK (Figure 2(E)), and SELENOT (Figure 2(G)) was down-regulated about 10% by HG. The mRNA expression levels of SELENOS and SELENON were down-regulated significantly of 23% (Figure 2(C)) and 35% (Figure 2(D)) by HG stimulation. Since SELENON and SELENOS can be down-regulated under HG stimulation which can activate TGF-β1 and induce the ECM production such as fibronectin, this result suggested that ER-resident selenoproteins, especially SELENOM, SELENOS, and SELENON involved in the progress of fibronectin expression and provoked us to do further experiments to find out the role of ER-resident selenoproteins in the progression of HMCs pathological change.

### Glucose and TGF-β1 down-regulate SELENOS and SELENON expression in a time-dependent manner

**Figure 3.**
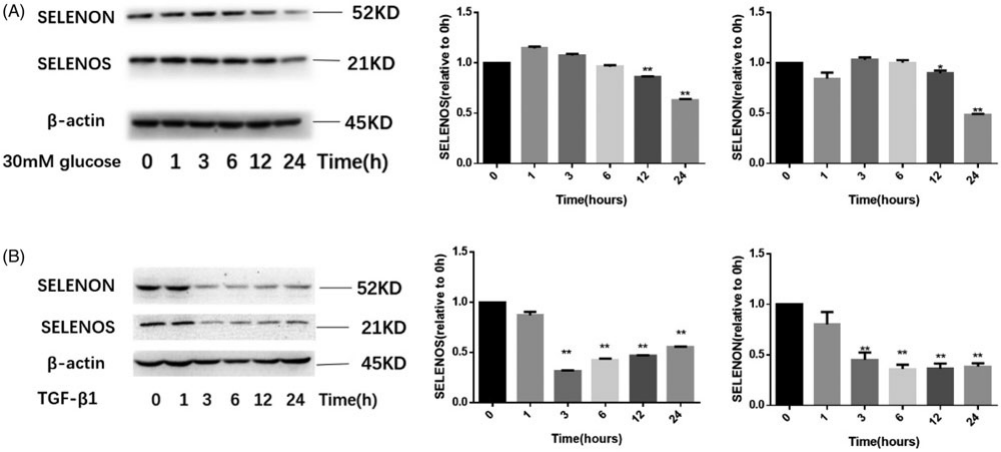
Glucose and TGF-β1 down-regulate SELENON and SELENOS expression in a time-dependent manner. HMCs were stimulated with HG (30 mM glucose) or TGF-β1 (10 ng/mL) for different time points. Then cell total protein was extracted and the expression of SELENOS and SELENON was detected by western-blotting. (A) The expression of SELENON and SELENOS under the stimulation of HG at indicated time points (0, 1, 3, 6, 12, and 24 h). (B) The expression of SELENON and SELENOS under the stimulation of TGF-β1 at indicated time points (0, 1, 3, 6, 12, and 24 h). HG: high glucose. *n* = 3, *p<.05 versus 0 h group, **p<.01 versus 0 h group.

Also, we set out to investigate whether TGF-β1 and HG regulate the expression of SELENON and SELENOS at protein level. Cells were stimulated with TGF-β1 (10 ng/mL) or HG (30 mM) for different times after 12 h synchronization, then total cell protein was extracted for western-blotting. As shown in Figure 3(A), both SELENON and SELENOS expression decreased significantly after 12 h HG stimulation, and reached the lowest point after 24 h stimulation. Similarly, after TGF-β1 stimulation of 3 h, the protein level expression of SELENON and SELENOS began to decrease and prolonging stimulation time cannot further reduce their expression amount (Figure 3(B)).

### Glucose and TGF-β1 down-regulate SELENON and SELENOS expression in a dose-dependent manner

**Figure 4.**
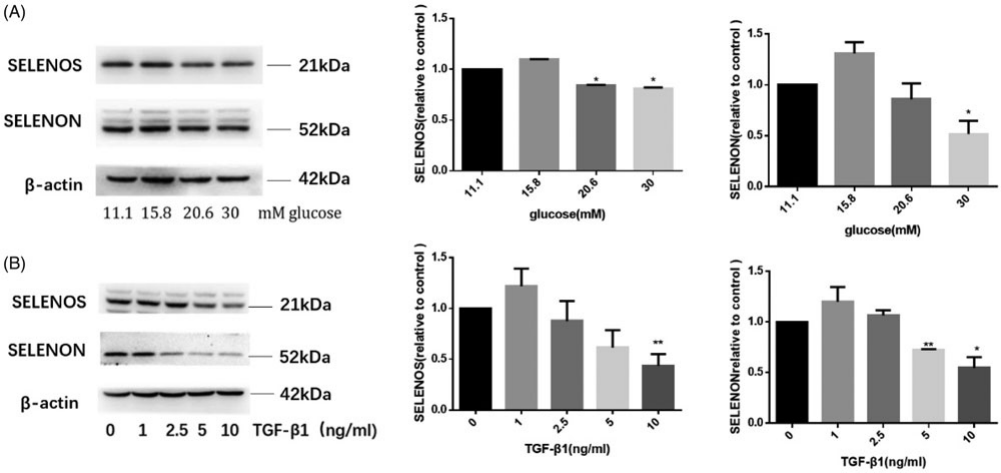
Glucose and TGF-β1 down-regulate SELENON and SELENOS expression in a dose-dependent manner. HMCs were stimulated with different concentration of glucose or TGF-β1 for 6 h. Then cell total protein was extracted and the expression of SELENOS and SELENON was detected by western blotting. (A) The expression of SELENON and SELENOS under the stimulation of glucose at the concentration of 11.1 mM, 15.8 mM, 20.6 mM, and 30 mM. (B) The expression of SELENON and SELENOS under the stimulation of TGF-β1 at the concentration of 0, 1, 2.5, 5, and 10 ng/mL, respectively. *n* = 3, *p<.05 versus control group, **p<.01 versus control group.

To further verify the regulation of TGF-β1 and glucose on SELENON and SELENOS in HMCs, also, we stimulated the cells with different glucose concentration (11.1 mM, 15.8 mM, 20.6 mM, and 30 mM) for 24 h or TGF-β1 (1, 2.5, 5, and 10 ng/mL) for 6 h. As shown in Figure 4(A), with the increase of glucose concentration, the expression of SELENON and SELENOS became lower and lower. Thus, glucose down-regulated the SELENON and SELENOS expression in a dose-dependent manner. Likewise, TGF-β1 also down-regulated the SELENON and SELENOS expression in a dose-dependent manner (Figure 4(B)). Those data further proved that glucose and TGF-β1 down-regulated the expression of SELENON and SELENOS significantly.

### Knocking down SELENOS expression by siRNA down-regulates TGF-β1-induced fibronectin expression

**Figure 5.**
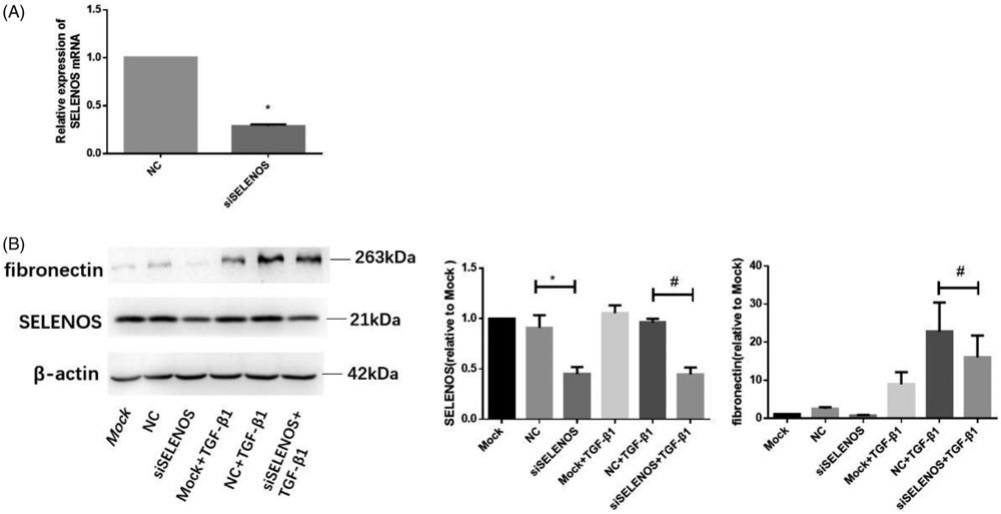
Knocking down SELENOS expression by siRNA down-regulates TGF-β1 induced fibronectin expression. HMCs were transfected with no sequence (mock group) or 50 nM NC sequence (NC group) or 50 nM siRNA sequence against SELENOS (siSELENOS group) for 24 h. After 12 h synchronization, cells were treated with or without TGF-β1 (10 ng/mL) for another 24 h. Total protein was extracted and the expression of SELENOS and fibronectin was detected by western-blotting. The data show the expression of SELENOS and fibronectin of each group. *n* = 3, *p<.05 versus NC group, ^#^*p*<.05 versus NC + TGF-β1 group.

Further experiments were performed to determine whether ER-resident selenoproteins regulate the fibronectin expression induced by TGF-β1. We chose SELENOS as the representative of the ER-localized selenoprotein for the next experiment. HMCs were transfected with no sequence (mock group) or 50 nM NC sequence (NC group) or 50 nM siRNA sequence against SELENOS (siSELENOS group) for 24 h. After 12 h synchronization, cells were treated with or without TGF-β1 (10 ng/mL) for another 24 h. Total protein was extracted and the expression of SELENOS and fibronectin was detected by western-blotting. Compared with NC group, the expression amount of SELENOS in the siSELENOS group decreased about 60% (Figure 5). Compared with the NC + TGF-β1 group, the expression of fibronectin decreased significantly in siSELENOS + TGF-β1 group (Figure 5). Those data suggest that knocking down SELENOS down-regulates TGF-β1-induced fibronectin expression.

## Discussion

In summary, we established HG or TGF-β1 induced HMCs fibronectin expression model *in vitro*. Then, expression of seven ER-resident selenoproteins was detected under HG conditions and we found SELENOS, SELENON were significantly down-regulated but SELENOM was significantly up-regulated in transcription level. Furthermore, we verified at the protein level that TGF-β1 and HG down-regulate the expression of SELENOS and SELENON, respectively. Finally, SELENOS was knocked down *in vitro* and we found that knocking down SELENOS decreased TGF-β1-induced fibronectin expression.

In the ER stress event induced by tunicamycin or carotene, the expression of SELENOS was significantly increased in cells [14]. Contrary to what is generally believed to be the anti-ER stress effect of selenoproteins, we found that stimulating HMCs with HG and TGF-β1 could down-regulate the expression of SELENOS and SELENON rather than up-regulating them. Consistent with our experimental results, Gao et al. [15] reported that glucose deprivation increased SELENOS gene expression on cultured HepG2 liver cancer cells. Walder et al. [16] showed that glucose inhibited SELENOS expression in a concentration-dependent manner *in vitro* cultured C2C12 muscle cells and 3T3-L1 adipocytes. *In vivo*, SELENOS expression in liver negatively correlated with blood glucose and serum insulin levels in type 2 diabetes mellitus and metabolic syndrome animal models [16]. Together, SELENOS and SELENON seem to be regulated by HG and TGF-β1 and are dysregulated in the stress state. At the moment, there is no research on the regulation effects of glucose or TGF-β1 on SELENON expression. Thus, we first report the regulation effects of glucose or TGF-β1 on SELENON.

Current researches on SELENOS are mainly focused on metabolic diseases and cardiovascular diseases [16,17]. And researches on SELENON mainly focus on myopathies [13]. To date, only these two of ER-resident selenoproteins have been involved in human disease. But there are still no reports of SELENOS on kidney disease or fibrosis in other tissues. Thus, we want to use SELENOS as a starting point for revealing the relationship between ER-resident selenoproteins and renal fibrosis. To explore whether down-regulating of ER-resident SELENOS by HG and TGF-β1 on HMCs effect on renal fibrosis, we knocked down SELENOS by SiRNA and found reduced SELENOS mRNA could decrease fibronectin expression on HMCs, which indicates that down-regulating of ER-resident SELENOS plays protective effect on renal fibrosis. Our result was contradictory to the results of most non-renal related experiments which suggest over-expression of SELENOS can play its role of anti ER-stress and antioxidant protection [13,14,18,19].

Based on existing research, it seems that SELENOS plays different roles in different cells, tissues, and organs. Speckmann et al. inhibited SELENOS expression in three colon cancer cell lines and the results showed ER stress markers did not increase even in the presence of the ER stress inducer [20]. Recent study also reported that no enhancement of gene markers of ER stress was observed in palmitate-treated myotubes rather than myoblasts response to SELENOS knockdown [21]. Combined with our finding, the major reason for these inconsistent results may be the difference in the types of studied cells. SELENOS also has different biological functions in different tissues and organs, even has opposite effects. In the pancreas and blood vessels, SELENOS can exert antioxidant protection and has anti-ER stress effects, while it promotes the occurrence and development of insulin resistance in the liver, adipose tissue, and skeletal muscle [22]. Even in the same tissue, Yao et al.’s study showed that relative distribution of ER-resident selenoprotein gene mRNA amounts and their responses to dietary Se deficiency were different in different skeletal muscle [23]. It was suggested that the participation of SELENOS in the regulation of ER stress was cell, tissue, and organ specific.

Our research first observed the attenuation effect of knocking down SELENOS on the fibronectin expression *in vitro*. Although this is a preliminary study, it indicates the value of this class of selenoproteins on renal fibrosis. Current understanding of SELENOS specific expression and function is limited. We consider that SELENOS knockdown alleviating the pathological state of HMCs is not through decreasing the production of ROS, but through regulating the activation of downstream signal molecule in TGF-β1 signal pathway. Hence, role of SELENOS played here is a signaling molecule, not a regulatory factor. Whether the human SELENOS gene promoter region has negative regulatory elements that can regulate SELENOS gene expression and whether there are TGF-β1 downstream signaling molecules such as Smad that can interact with these elements should also be in-depth explored. Further research should also be performed on kidney disease animal models and reveal the mechanism of their effects to fully utilize the advantages of SELENOS and weaken its adverse effects on kidney fibrosis.
